# Tumor-suppressive miR-3650 inhibits tumor metastasis by directly targeting NFASC in hepatocellular carcinoma

**DOI:** 10.18632/aging.101981

**Published:** 2019-06-04

**Authors:** Jian Wu, Wen-Jin Huang, Hong-Li Xi, Ling-Yun Liu, Shu-Tong Wang, Wen-Zhe Fan, Bao-Gang Peng

**Affiliations:** 1 Department of Hepatic Surgery, The First Affiliated Hospital of Sun Yat-sen University, Guangzhou 510080, China; 2 Department of Radiotherapy Oncology, Affiliated Cancer Hospital & Institute of Guangzhou Medical University, Guangzhou 510095, China; 3 Department of Clinical Laboratory, Affiliated Cancer Hospital & Institute of Guangzhou Medical University, Guangzhou 510095, China; 4 Department of Hepatobiliary and Pancreatic Surgery, Affiliated Hospital of Guilin Medical University, Guilin 5410000, China; 5 Department of Interventional Oncology, The First Affiliated Hospital of Sun Yat-sen University, Guangzhou 510080, China

**Keywords:** hepatocellular carcinoma, miR-3650, NFASC, metastasis, EMT

## Abstract

In recent years, a growing body of evidence has provided support for the important role of microRNAs (miRNAs) in the progression of human cancers. A recent study showed that a novel miRNA miR-3650 expression was significantly decreased in hepatocellular carcinoma (HCC). However, the precise role of miR-3650 in HCC have remained poorly understood. In this study, we found that miR-3650 expression was frequently decreased in HCC tissues. Low expression of miR-3650 is positively associated with tumor metastasis and poor survival of HCC patients. Forced expression of miR-3650 significantly inhibited the migration and epithelial-mesenchymal transition (EMT) of HCC cells. Through bioinformatic analysis and luciferase assays, we confirmed that neurofascin (NFASC) is a directly target mRNA of miR-3650. Rescue experiment demonstrated that NAFSC overexpression could partially counteracted the inhibitory effect of miR-3650 in HCC metastasis and EMT. In conclusion, our findings are the first time to demonstrate that reduced expression of miR-3650 in HCC was correlated with tumor metastasis and poor survival. MiR-3650 repressed HCC migration and EMT by directly targeting NFASC. Our findings suggested that miR-3650 may serve as a potential prognostic marker and promising application in HCC therapy.

## Introduction

Hepatocellular carcinoma (HCC) is the sixth most frequent cancer and the fourth most common cause of cancer-related death worldwide in 2018 [1]. About 80% HCC cases occur in East Asia (including China) and Sub-Saharan Africa, where the main risk factors are chronic hepatitis B and aflatoxin B1 exposure [2]. Although great developments in the diagnosis and therapy of this disease in the past decades, the prognosis of HCC patients is still dismal [3,4]. Moreover, most newly diagnosed HCC patients are unresectable distant metastasis. The median overall survival (OS) of these patients treated with transcatheter arterial chemoembolization or sorafenib was less than one year because of the high rate of recurrence and metastasis [5,6]. In clinical practice, many HCC patients with same clinical stage have obviously different outcome, suggesting the inherent heterogeneity of the biological behavior of cancer cells [7]. Therefore, a better understanding of the underlying mechanisms involved in HCC metastasis will provide potential molecular targets for treatment of HCC metastasis and improve the prognosis of HCC patients.

As the development of high-throughput transcriptome analysis and next generation sequencing, it has been recognized that up to 90% of genomic DNA are not transcribed and translated into proteins [8], but they can be transcribed into noncoding RNAs [9]. MiRNAs are a class of 18-22 nucleotide small noncoding RNAs that can negatively regulate target genes expression by partially base-pairing with the 3 untranslated regions (3’-UTR) of target mRNAs [10]. Up to date, over 38,000 miRNAs have been identified and play key roles in a large range of biological processes including regulation of cell proliferation, apoptosis, stress resistance, epithelial-mesenchymal transition (EMT) and metastasis [11,12]. Recent studies have shown the alterations in miRNAs expression during the progression of HCC, highlighting their potentials for the diagnostic and prognostic applications, and targeted therapy for this malignancy [13]. For instance, it has been found that miR-214-5p overexpression inhibited the migration and EMT by targeting Wiskott-Aldrich Syndrome Like in HCC cells [14]. And miR-135a promoted the metastasis of HCC cells by inhibiting tumor suppressor forkhead box O1 (FOXO1) [15].

A recent study showed that compared with non-side population (SP) cells, miR-3650 expression was significantly reduced in HCC SP cells [16]. This finding suggested that miR-3650 may play critical role in the progression of HCC. However, the biological functions and molecular mechanisms of miR-3650 in HCC remain mysterious. In this study, we investigated the expression and prognostic value of miR-3650 in HCC patients. We further assessed the effect of miR-3650 on HCC migration and EMT in vitro. Finally, we explored the underlying mechanism of miR-3650 in HCC metastasis.

## RESULTS

### The expression of miR-3650 is significantly decreased and correlated with clinical stage in HCC patients

**Figure 1:**
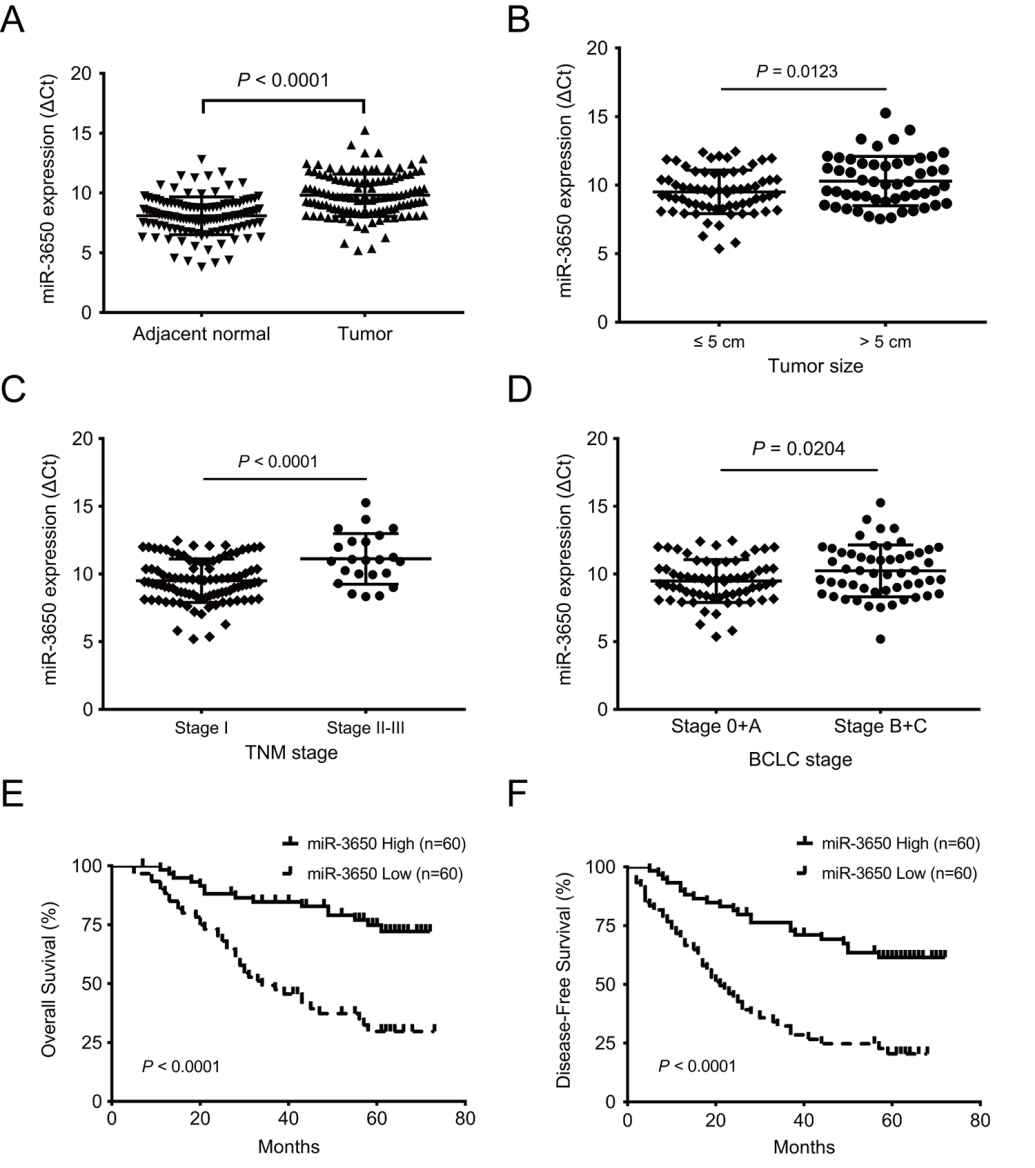
**Reduced expression of miR-3650 in HCC tissues is correlated with poor prognosis of HCC patients.** (**A**) Relative expression of miR-3650 between HCC tissues and adjacent normal liver tissues by RT-PCR. Results were presented as Δcycle threshold (ΔCt) in tumor tissues relative to adjacent normal tissues. (**B**) The correlation between miR-3650 expression and tumor size: ≤ 5 cm and > 5 cm. (**C**) The correlation between miR-3650 expression and TNM stage: stage I and stage II-III according to the latest AJCC (American Journal of Critical Care) guide. (**D**) The correlation between miR-3650 expression and Barcelona Clinic Liver Cancer (BCLC) stage: stage 0+A and stage B+C. (**E**) and (**F**) Kaplan-Meier plots of overall-survival and disease-free survival in HCC patients with high (n=60) and low (n=60) levels of miR-3650. Low expression of miR-3650 was associated with reduced survival of HCC patients. (all *P* < 0.0001).

To determine the importance of miR-3650 in HCC patients, we first detected the expression level of miR-3650 in 120 paired HCC samples by quantitative RT-PCR. The results showed that compared with adjacent normal liver tissues, miR-3650 expression in HCC tissues was significantly decreased (*P* < 0.0001, Fig. 1A). Moreover, miR-3650 expression was negatively correlated with tumor size, TNM stage (tumor-node-metastasis stage) and Barcelona Clinic Liver Cancer (BCLC) stage (all *P* < 0.05, Fig. 1B-1D). To evaluate the clinical relevance of miR-3650 in HCC patients, the median value of miR-3650 expression (0.315) was defined as a cutoff value to divide HCC patients into high- or low-expression groups. The relationship between clinicopathological parameters and miR-3650 expression was summarized in Table 1. Reduced expression of miR-3650 was remarkably associated with tumor size (*P*=0.010), TNM stage (*P*=0.037) and BCLC stage (*P*=0.017). In contrast, miR-3650 expression displayed no correlation with other clinicopathological variables (Table 1). These results suggested that reduced expression of miR-3650 might be involved in the progression and metastasis of HCC.

**Table 1:** The correlation between clinicopathological parameters and miR-3650 expression levels in hepatocellular carcinoma patients.

Characteristics	No of patients	LINC01939 Expression (%)		*P*-value ^a^
		Low	High	
Gender				
Female	15	6 (40.0%)	9 (60.0%)	0.408
Male	105	54 (51.4%)	51 (48.6%)	
Age (years)				
≤ 50	63	31 (49.2%)	32 (50.8%)	0.855
> 50	57	29 (50.9%)	28 (49.1%)	
Tumor size (cm)				
≤ 5.0	68	27 (39.7%)	41 (60.3%)	0.010
> 5.0	52	33 (63.5%)	19 (36.5%)	
AFP (ng/ml)				
≤ 400	71	33 (46.5%)	38 (53.5%)	0.353
> 400	49	27 (55.1%)	22 (44.9%)	
HBsAg				
Negative	9	6 (66.7%)	3 (33.3%)	0.491
Positive	111	54 (48.6%)	57 (51.4%)	
GGT (U/L)				
≤ 50	71	34 (47.9%)	37 (52.1%)	0.577
> 50	49	26 (53.1%)	23 (46.9%)	
Liver cirrhosis				
No	20	9 (45.0%)	11 (55.0%)	0.624
Yes	100	51 (51.0%)	49 (49.0%)	
Satellite nodule				
No	104	49 (47.1%)	55 (52.9%)	0.178
Yes	16	11 (68.8%)	5 (31.2%)	
Vascular invasion				
No	99	47 (47.5%)	52 (52.5%)	0.230
Yes	21	13 (61.9%)	8 (38.1%)	
Tumor differentiation				
I-II	81	40 (49.4%)	41 (50.6%)	0.845
III-IV	39	20 (51.3%)	19 (48.7%)	
TNM stage				
I	97	44 (45.4%)	53 (54.6%)	0.037
II-III	23	16 (69.6%)	7 (30.4%)	
BCLC stage				
0-A	67	27 (40.3%)	40 (59.7%)	0.017
B+C	53	33 (62.3%)	20 (37.7%)	

^a^ *P* < 0.05, Chi-square test. GGT, gamma-glutamyltransferase; AFP, α-fetoprotein; TNM stage, tumor-node-metastasis stage; BCLC stage, Barcelona Clinic Liver Cancer stage.

### Low expression of miR-3650 predicts poor prognosis in HCC patients

Since miR-3650 was negatively associated with tumor size and clinical stage of HC patients, we reasoned that miR-3650 is correlated with patients’ outcome. As expected, survival analysis indicated that patients with miR-3650 low-expression have apparently shorter 5-year OS (29.7% vs. 74.8%) and 5-year disease-free survival (DFS) (20.4% vs. 61.5%) than those with high-expression (all *P* < 0.0001, Fig. 1E and 1F). To figure out the prognostic value of miR-3650 in this disease, univariate and multivariate analyses were performed on clinicopathological variables and patient survival. The univariate Cox proportional analysis indicated that tumor size, satellite nodule, vascular invasion and miR-3650 expression are significantly prognostic predictors for OS and DFS (Table 2). Multivariate Cox proportional analysis further demonstrated that miR-3650 was an independent protective predictor for OS and DFS in HCC patients (all *P* < 0.0001, Table 2). In addition, vascular invasion also was an independent risk factor for OS and DFS (all *P* < 0.0001), and AFP level also was an independent prognostic factor only for OS (*P*=0.009). These above results suggested that miR-3650 may serve as a potential prognostic biomarker for HCC patients.

**Table 2:** Univariate and multivariate analyses of various potential prognostic factors in HCC patients.

Variables*	OS			DFS		
	Univariate *P* value	Multivariate analysis		Univariate *P* value	Multivariate analysis	
		*P* value	HR (95% CI)		*P* value	HR (95% CI)
Gender (Male vs. Female)	0.630			0.534		
Age, years (> 50 vs. ≤ 50)	0.955			0.340		
Tumor size (> 5cm vs. ≤ 5cm)	0.001	0.636	1.168 (0.613-2.226)	0.005	0.971	0.990 (0.568-1.724)
AFP (ng/ml) (> 400 vs. ≤ 400)	0.009	0.009	2.060 (1.198-3.542)	0.104		
HBsAg (Positive vs. Negative)	0.998			0.271		
GGT (U/L) (> 50 vs. ≤ 50)	0.211			0.126		
Liver cirrhosis (Yes vs. No)	0.113			0.088		
Satellite nodule (Yes vs. No)	0.010	0.430	1.326 (0.659-2.668)	0.024	0.302	1.434 (0.723-2.843)
Vascular invasion (Yes vs. No)	< 0.001	< 0.001	6.990 (3.492-13.993)	< 0.001	< 0.001	7.138 (3.752-13.581)
Tumor differentiation (III-IV vs. I-II)	0.412			0.688		
miR-3650 (high vs. low)	< 0.001	< 0.001	0.266 (0.144-0.492)	< 0.001	< 0.001	0.296 (0.174-0.504)

* Because TNM stage and BCLC stage was combined with multiple clinical variables such as tumor size, number and tumor thrombus; we did not enter the TNM stage and BCLC stage into univariate and multivariate analyses to avoid any bias in analysis.

GGT, gamma-glutamyltransferase; AFP, α-fetoprotein; OS, overall survival; DFS, disease-free survival; HR, hazard ratio; CI, confidence interval.

### Overexpression of miR-3650 inhibits HCC cells metastasis and EMT in vitro

**Figure 2:**
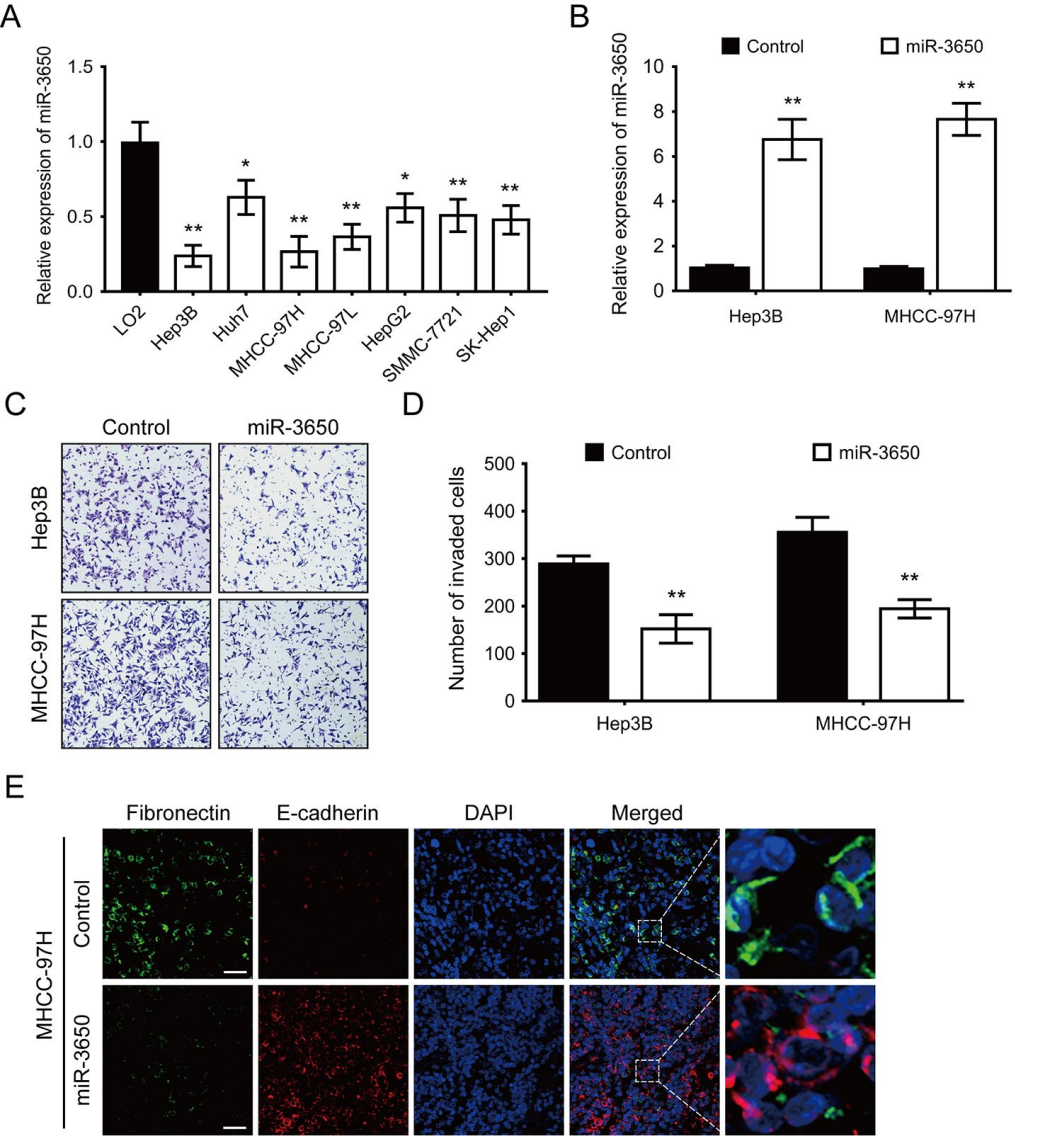
**Forced expression of miR-3650 inhibits HCC migration and EMT in vitro.** (**A**) Relative expression of miR-3650 in HCC cell lines (Hep3B, Huh7, MHCC-97H, MHCC-97L, HepG2, SMMC-7721 and SK-Hep1) compared with that of the immortalized liver cell line LO2. Data was presented as expression fold change relative to LO2. (**B**) RT-PCR analysis of miR-3650 mRNA levels in Hep3B and MHCC-97H cells transfected with miR-3650 mimics or control mimics. (**C**) and (**D**) Representative images and quantification of migration of Hep3B and MCHH-97H cells after miR-3650 overexpression by transwell assays. (**E**) Representative images of immunofluorescence micrographs of the localization and expression of Fibronectin (green) and E-cadherin (red). Nuclei were counterstained with DAPI (blue). Scale bars represent 50 μm. For all quantitative results, the data are presented as the mean ± SD from three independent experiments. * *P* < 0.05 and ** *P* < 0.01.

We also performed RT-PCR to determine the expression of miR-3650 in seven HCC cell lines and one immortalized liver cell line LO2. The result showed that miR-3650 expression was generally lower in HCC cell lines than LO2 (Fig. 2A). The significant decrease of miR-3650 expression in HCC tissues and cells prompted us to observe its biological significance in HCC. In order to manipulate miR-3650 expression, Hep3B and MHCC-97H cells with the lowest level of miR-3650 were transfected with miR-3650 mimic, and effective overexpression in both cell lines were verified by RT-PCR (Fig. 2B). Transwell assay were subsequently conducted, and the result indicated that enforced expression of miR-3650 significantly inhibited HCC cell migratory capacity (Fig. 2C and 2D). Moreover, we did immunofluorescence (IF) assay to investigate whether miR-3650 could affect epithelial-mesenchymal transition (EMT) in HCC cells. To our interest, miR-3650 overexpression increased the expression of epithelial marker E-cadherin while decreased the expression of mesenchymal marker Fibronectin (Fig. 2E). We also found that miR-3650 overexpression promoted the morphology of MHCC-97H from the dispersed type into the condensed type (Fig. 2E). Taken together, our data demonstrated that miR-3650 overexpression could suppress HCC migration and EMT in vitro.

### NFASC is identified as a directly target mRNA of miR-3650 in HCC

**Figure 3:**
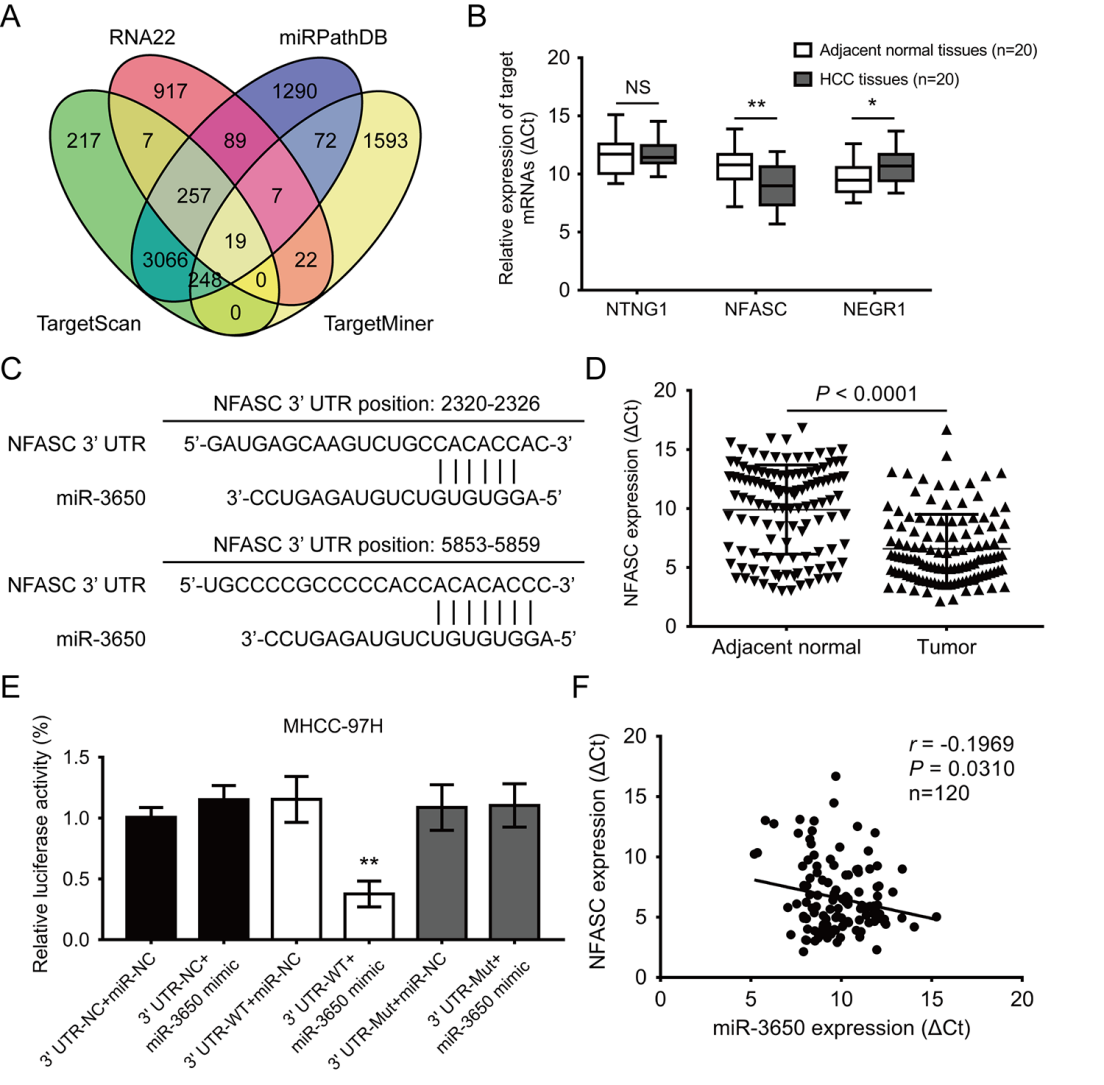
**NFASC is a direct target of miR-3650 in HCC.** (**A**) Venn diagrams showing the number of potential targeted mRNAs of miR-3650 from four databases: TargetScan, RNA22, miRPathDB and TargetMiner. (**B**) RT-PCR analysis of the expression of three candidate mRNAs in 20 paired HCC tissues and adjacent normal tissues. (**C**) Predicted binding sites of miR-3650 and NFASC mRNA 3’-UTR as predicted by the Targetscan algorithm. (**D**) Relative expression of NFASC mRNA between 120 paired HCC tissues and adjacent normal liver tissues by RT-PCR. (**E**) Relative luciferase activities of wild type (WT) and mutated (Mut) NFASC mRNA 3’-UTR reporter in MHCC-97H cells co-transfected with miR-3650 mimic. (**F**) The correlation between miR-3650 and NFASC expression in HCC tumor tissues. Error bars: Means ± SD (n=3). * *P* <0.05 and **P < 0.01 versus control cells.

It is well known that miRNAs directly bind to the 3’-UTR region of protein coding genes, and result in either transcriptional inhibition or mRNA degradation [17]. Thus, we predicted potential target mRNAs of miR-3650 using four online publicly available algorithms (TargetScan, RNA22, miRPathDB and TargetMiner). The result showed 19 mRNAs were the predicted targets of miR-3650 (Fig. 3A). We further predicted the signaling pathway of 19 potential targeted mRNAs using the online database DAVID 6.7. As shown in Fig. S1, gene Ontology KEGG pathway enrichment analysis indicated that these predicted mRNAs were only enriched in cell adhesion molecules, which included netrin G1 (NTNG1), neurofascin (NFASC) and neuronal growth regulator 1 (NEGR1). Then we further confirmed that NFASC expression was significantly increased in 20 paired HCC tissues compared with adjacent normal tissues (Fig. 3B). Figure 3C displayed tow putative binding sites within miR-3650 and 3’-UTR of NFASC predicted using TargetScan 7.2 algorithm. In addition, we also found that the expression of NFASC was significantly higher in HCC tissues than in adjacent normal tissues (Fig. 3D).

To verify the direct binding relationship between miR-3650 and NFASC, we subcloned wide-type (NFASC 3’-UTR-WT) or mutated (NFASC 3’-UTR-Mut) miR-3650 binding site into the downstream of the dual-luciferase reporter gene in the pmirGLO-basic vector. Luciferase activity assay demonstrated that co-transfection of pmirGLO-NFASC 3’-UTR-WT with miR-3650 mimic resulted in lower luciferase activity in MHCC-97H cells (Fig. 3E). Meanwhile, we investigated that co-transfection of pmirGLO-NFASC-3’-UTR-Mut with miR-3650 or miR-NC both did not strikingly change in relative luciferase activity (Fig. 3E). As expected, there was a negative relationship between miR-3650 and NFASC expression in 120 HCC tissues (*r* = −0.1969, *P*< 0.0310, Fig. 3F). Primed by these findings, these results provided evidence that miR-3650 directly target 3’-UTR regions of NFASC gene in HCC.

### MiR-3650 inhibits HCC cells migration and EMT via targeting NFASC

**Figure 4:**
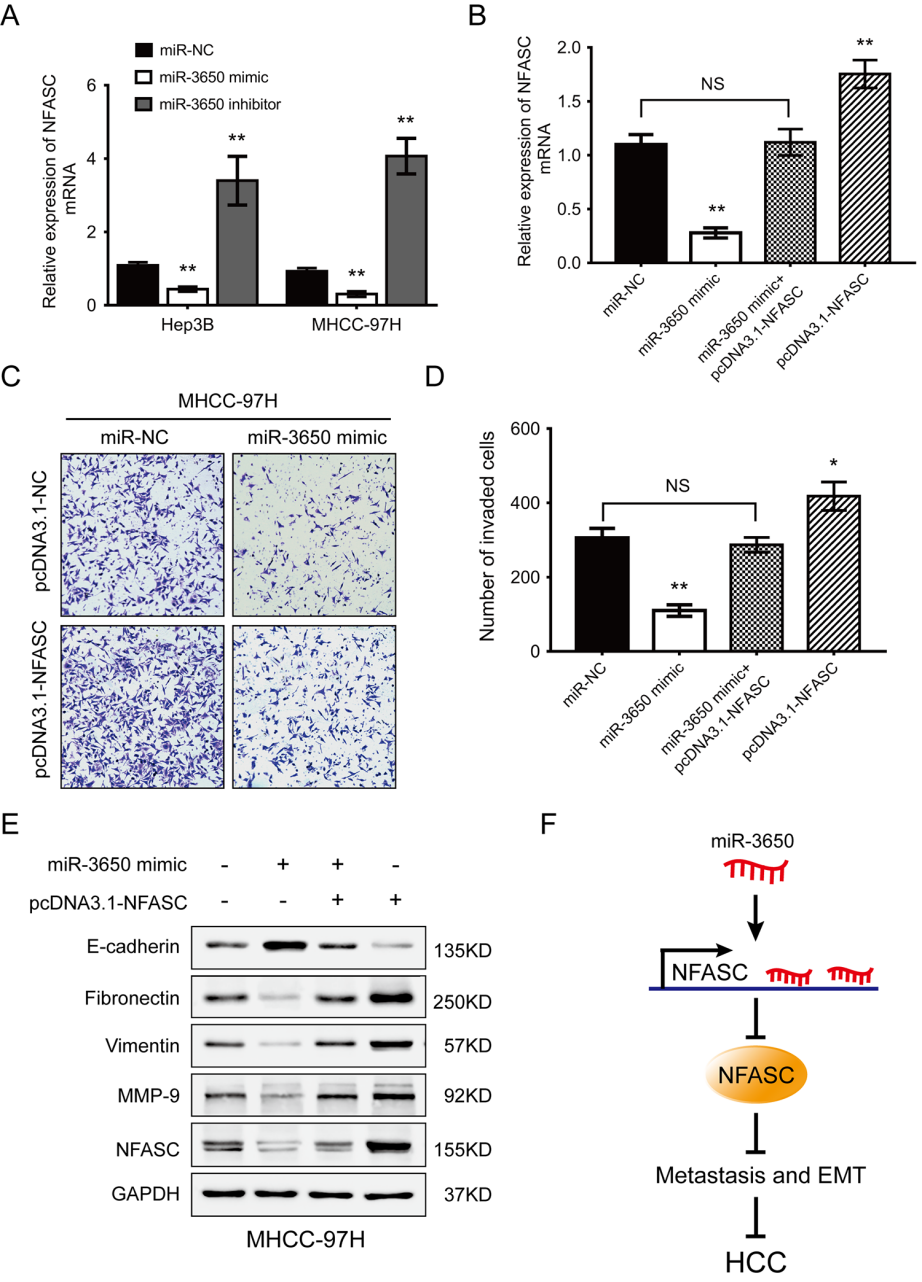
**NFASC expression mediates the anti-metastatic effects of miR-3650 in HCC cells.** (**A**) RT-PCR analysis confirmed that ectopic miR-3650 expression decreased NFASC expression while inhibition of miR-3650 increased NFASC expression. (**B**) NFASC mRNA level in MHCC-97H cells following overexpression of miR-3650 and/or NFASC expression vector lacking the 3’-UTR. Transwell assay of MHCC-97H cells after overexpression of miR-3650 and/or co-transfected with miR-NFASC expression vector lacking the 3’-UTR. Representative images (**C**) and quantifications (**D**) were shown. Error bars: mean ± SD (n=3). NS, no significant, * *P* <0.05 and ** *P* <0.01. (**E**) Western blots assay of the expression of EMT-related proteins (E-cadherin, Fibronectin, Vimentin and MMP-9 expression) and NFASC protein in MHCC-97H cells after transfection with miR-3650 mimic and/or pcDNA3.1-NFASC vector. (**F**) Schematic diagram of dysregulated miR-3650/NFASC axis in the inhibition of HCC metastasis.

To explore whether the anti-metastatic effect of miR-3650 were mediated through targeting NFASC, gain-of-function rescue experiments were performed. Firstly, we found that NFASC mRNA expression was significantly reduced or enhanced after Hep3B and MHCC-97H cells were transfected with miR-3650 mimic or inhibitor, respectively (Fig. 4A). Secondly, MHCC-97H cells co-transfected with miR-3650 mimic and pcDNA3.1-NFASC partially restored the inhibitory effect of miR-3650 mimic only (Fig. 4B). Similarly, transwell assay indicated that the suppressive effect of miR-3650 on HCC migration could be partially rescued by co-transfected with pcDNA3.1-NFASC, which contained the coding sequences but lacked the 3’-UTR regions of NFASC (Fig. 4C and 4D). As EMT plays critical roles in cancer metastasis [18], we further performed western blot assay to observe the expression of EMT-related proteins. The results showed that miR-3650 led to decreased expression of E-cadherin and elevated expression of Fibronectin, Vimentin and MMP-9 (Fig. 4E), which was consistent with the result of Fig. 2E. As anticipated, the abnormal expression of EMT-related proteins induced by miR-3650 mimic was contracted after cells co-transfected with pcDNA3.1-NFASC (Fig. 4E). Collectively, these data suggest that miR-3650 inhibits HCC cells migration and EMT in a NFASC-dependent manner.

## DISCUSSION

The progression of HCC is a complex multistep process that involves huge genomic alterations and epigenetic modifications [19]. Unfortunately, none of the molecular classification of HCC so far can predict the prognosis and metastasis of this malignancy in clinical practice [20]. Considerable evidences demonstrated the complicated functional involvement of dysregulated miRNAs in the carcinogenesis, progression, recurrence, metastasis and survival of HCC cases [21]. However, the exact molecular mechanism of dysregulated miRNAs in HCC metastasis has remained elusive.

As a novel miRNA, the precursor of miR-3650 gene located in chromosome 5. Recently, miR-3650 has been reported to be involved in few cancer types. Two studies showed that miR-3650 expression was reduced in laryngeal carcinoma tissues and colorectal cancer cell [22,23]. More recently, Xie et al. indicated that miR-3650 was significantly decreased in HCC SP cells compared with non-SP cells [16], suggesting that miR-3650 may play an important role in HCC stemness and progression. Whereas no studies have reported the expression pattern, biological function and underlying mechanism of miR-3650 in HCC. In the current study, miR-3650 expression was significantly decreased in HCC tissues, which was similar to the previous study [16]. Low expression of miR-3650 was positively associated with adverse prognosis of HCC patients and could be used as an independent prognostic predictor. Moreover, our study is the first report that miR-3650 overexpression inhibited HCC migration and EMT processes in vitro. Our results provide a more comprehensive understanding of the tumor suppressor role of miR-3650 in HCC metastasis.

It has been demonstrated that the interaction between miRNAs and 3’-UTR of their target mRNAs through complementary base pairing exerts their translational repression and target mRNAs degradation, then influences cell fate and biological functions [24,25]. Previous study has demonstrated that miR-487 enhanced HCC cells metastasis by directly binding to sprouty-related EVH1 domain containing 2 induced MAPK pathway [26]. This prompted us to investigate the downstream target mRNAs of miR-3650 in HCC. In this study, we confirmed that NFASC is the directly target of miR-3650. Overexpression of NAFSC could partially counteracted the inhibitory effect of miR-3650 in HCC metastasis and EMT. Actually, Samulin Erdem et al. showed that knockdown of NFASC could decrease the migration of non-small cell lung cancer cells, rearrange the actin cytoskeleton and inhibit F-actin networks [27], which was consistent with our current results. This implying that miR-3650 inhibit the migration and EMT abilities of HCC cells at least in part by directly binding and inhibiting NFASC expression (Fig. 4F).

Interestingly, mounting reports have verified that many miRNAs expression were directly regulated by long noncoding RNAs (lncRNAs) as upstream regulators in several human cancers. LncRNAs usually function as competitive endogenous RNA (ceRNA) or molecular sponges for miRNAs [28]. A recent study showed that LINC01133 inhibited the EMT and metastasis of gastric cancer cells by acting as a ceRNA for miR-106a-3p to inhibit the adenomatous polyposis coli gene expression [29]. However, the upstream regulator of miR-3650 in HCC remains unanswered, and whether miR-3650/NFASC axis inhibits HCC metastasis by regulating certain signaling pathways has not yet been unraveled. Further investigations were made to solve these issues in our future studies.

In conclusion, our study reported the following new findings: (1) miR-3650 expression is significantly reduced in HCC tissues and cells; (2) low expression of miR-3650 is positively associated with poor survival of HCC patients and could be used as an independent prognostic predictor; (3) miR-3650 functions as a tumor suppressor in the migration and EMT abilities of HCC; (4) NFASC is a directly target mRNA of miR-3650; (5) miR-3650 inhibits HCC cells migration and EMT by binding and inhibiting NFASC. Our study underscores the inhibitory role of miR-3650 in HCC metastasis and facilitate the development of miRNA-direct prognosis and therapeutics against HCC patients.

## MATERIALS AND METHODS

### HCC patients and samples collection

This study was approved by the Institutional Review Board and Human Ethics Committee of The First Affiliated Hospital of Sun Yat-sen University. Fresh-frozen cancer tissues and adjacent normal liver tissues were obtained from 120 HCC patients who received hepatectomy between February 2009 and December 2011 at our hospital. The eligibility criteria were as follows: (1) all the samples were pathologically diagnosed by two experienced pathologists; (2) none of our patients received any radiotherapy and/or chemotherapy before surgery; (3) no serious complications or other malignant diseases; (4) 18 to 75 years old; (5) written informed consent was obtained from all patients before surgery. Clinical stage was classified according to the 7th edition of tumor-node metastasis (TNM) classification of the American Joint Committee on Cancer Staging and the Barcelona Clinic Liver Cancer (BCLC) staging system. Overall survival was defined as the time from the date of surgery to the date of death from any cause or last date of follow-up; Disease-free survival is measured as the time from the date of surgery to the date of relapse, metastasis or last date of follow-up.

### HCC cell lines and culture conditions

HCC cell lines were obtained from the Cell Bank of the Chinese Academy of Sciences (Shanghai, China), including Hep3B, Huh7, MHCC-97H, MHCC-97L, HepG2, SMMC-7721 and SK-Hep1 and the immortalized human hepatocyte cell line LO2. These cell lines were cultured in DMEM (Life Technologies, Inc.) supplemented with 10% fetal bovine serum (FBS) at 37°C with 5% CO_2_.

### RNA extraction and quantitative real-time polymerase chain reaction analyses (RT-PCR)

Total RNA was isolated from fresh-frozen tissues and HCC cells using TRIzol reagent (Invitrogen, NY, USA) as per the manufacturer’s instructions. The quantity of RNA samples was measured by a NanoDropTM 1000 spectrophotometer (ThermoFisher Scientific, Waltham, MA, USA). 1 μg total RNA and All-in-one^TM^ First-Strand cDNA Synthesis Kit (GeneCopoeia, Rockville, USA) were used to generate cDNA, which was subjected to RT-PCR using All-in-One™ miRNA qRT-PCR Detection Kit (Gene-Copoeia, Carlsbad, CA, USA). PCR was performed on a Roche Light Cycler 480 instrument (Roche, Basel, Switzerland) according to a standard method as described previously [30]. The relative expression of miR-3650 was calculated using the 2^−ΔΔCt^ method and U6 was used as the endogenous control. The primer sequences used in this study are shown in supplementary Table S1.

### Lentivirus and cell transfection

Lentivirus particles containing miR-3650 mimic, inhibitor and scramble sequences were purchased from GenePharma (Shanghai, China). The Hep3B and MHCC-97H cells were transfected with these lentivirus particles. At 48h after transfection, cells were treated with puromycin (2 μg/ml) for 7 days to construct cell lines with stable miR-3650 knockdown or overexpression. NFASC ORF cDNA but lacked the 3’-UTR were amplified by PCR and then sub-cloned into the eukaryotic expression vector pcDNA3.1 (+), and the constructs were called pcDNA3.1-NFASC. The cell transfection was performed with Lipofectamine 3000 to construct HCC cells with stable NFASC overexpression.

### In vitro migration assay

The in vitro migration assay was performed with the Corning Polycarbonate Membrane Insert transwell chambers (Corning Costar Corp, Cambridge, MA, USA). The migration assay was conducted according to a previous report [31]. Briefly, HCC cells (5~6 × 10^5^) in serum-free media were seeded into the upper chamber (8-μm pore size). 600 μl medium containing 20% FBS was added to the lower chamber as the chemoattractant. After incubation at 37°C for 24h, cells were fixed in methanol, stained with 0.1% crystal violet. After scraping of cells remaining on the upper chamber by cotton swab, those migrated into the lower chamber were imaged and counted under the microscope.

### Immunofluorescence (IF) assay

HCC cells were seeded into 24-well culture plate with a glass coverslip over each well and allowed to attach overnight. The cells were washed with PBS and fixed with 4% paraformaldehyde for 20 min and then permeabilized with 0.25% Triton X-100 (Sigma) for 10 min. After blocked with 4% bovine serum albumin, the cells were incubated overnight at 4°C with rabbit anti-Fibronectin or anti-E-cadherin antibody (Cell Signaling Technology, Danvers, MA, USA). On the following day, the cells were incubated with secondary fluorescein isothiocyanate (FITC)-conjugated goat anti-rabbit antibody (Sigma-Aldrich, Poole, UK) for 1 h. HCC cells were then incubated with 4’,6-diamidin-2-phenylindole (DAPI; Life Technologies, Carlsbad, CA, USA) for 15 min to stain the nuclei. Images of the cells were acquired using a fluorescence microscope (FV1000; Olympus, Tokyo, Japan).

### Dual-luciferase reporter assay

The 3’-UTR of NFASC containing the putative binding sites of miR-3650 was amplified and inserted into the dual-luciferase reporter vector pmirGLO (Promega, Madison, WI, USA) to construct the reporter vector named as pmirGLO-NFASC-3’-UTR-WT. To confirm the direct relationship between miR-3650 and NFASC, we also construct mutant vectors with point mutations in the miR-3650-binding sites using a QuikChange Site-Directed Mutagenesis kit (Stratagene, La Jolla, CA, USA) and termed as pmirGLO-NFASC-3’-UTR-Mut. Dual-luciferase reporter assay was performed as described previously [32]. Briefly, HCC cells were seeded in a 48-well plate and then transfected with firefly luciferase plasmids, control Renilla luciferase vector pRL-TK, together with miR-3650 mimics or miR-NC and pmirGLO-NFASC-3’-UTR-WT or pmirGLOXIST--NFASC-3’-UTR-Mut. Luciferase activity levels were measured using the Dual-Luciferase Reporter Assay Kit (Promega, Madison, WI, USA) according to the manufacturer’s instructions.

### Western blot analysis

The procedure of Western blot was performed as previously described [33]. Briefly, HCC cells were collected and lysed in RIPA lysis buffer with protease inhibitor (Selleck, Houston, USA) on the ice. After quantification, 30 μg of lysate protein were separated by SDS-PAGE gel and then transferred onto PVDF membrane. After blocking nonspecific binding sites with 5% non-fat milk, the membranes were incubated with primary antibodies at 4°C overnight. The proteins in the membranes were visualized with the SuperSignal® ECL Kit (Pierce, USA). The primary antibodies used in this study included GAPDH, E-cadherin, Vimentin, Fibronectin (Cell Signaling Technology, USA), NFASC (Proteintech, Wuhan, China) and MMP-9 (Santa Cruz, USA). HRP-conjugated goat anti-rabbit or anti-mouse IgG antibody (Abcam, Cambridge, UK) was used as the secondary antibody. GAPDH was used as a loading control.

### Statistical analyses

Statistical analyses were performed using the SPSS 24.0 software (SPSS, Inc., Chicago, IL, USA) and GraphPad Prism V7 (GraphPad Prism, Inc., USA). For the experimental studies, our data are presented as mean ± standard deviation (SD). Comparisons between groups were analyzed by the two-tailed unpaired Student’s *t* test or one-way ANOVA. Kaplan-Meier plots and log-rank test were used to estimate cancer specific survival curves. *P* value of < 0.05 was considered to be statistically significant. Each experiment was carried out in repeated triplicates.

## SUPPLEMENTARY MATERIAL

Supplementary Figure and Table
