# Supplementary material for: Tumor-suppressive miR-3650 inhibits tumor metastasis by directly targeting NFASC in hepatocellular carcinoma
Source: Aging (Albany NY). 2019 Jun 4;11(11):3432–44. doi: 10.18632/aging.101981 (PMC6594810; doi:10.18632/aging.101981)
Supplement: Supplementary Figure and Table [file aging-11-101981-s001.pdf]

SUPPLEMENTARY MATERIAL

A

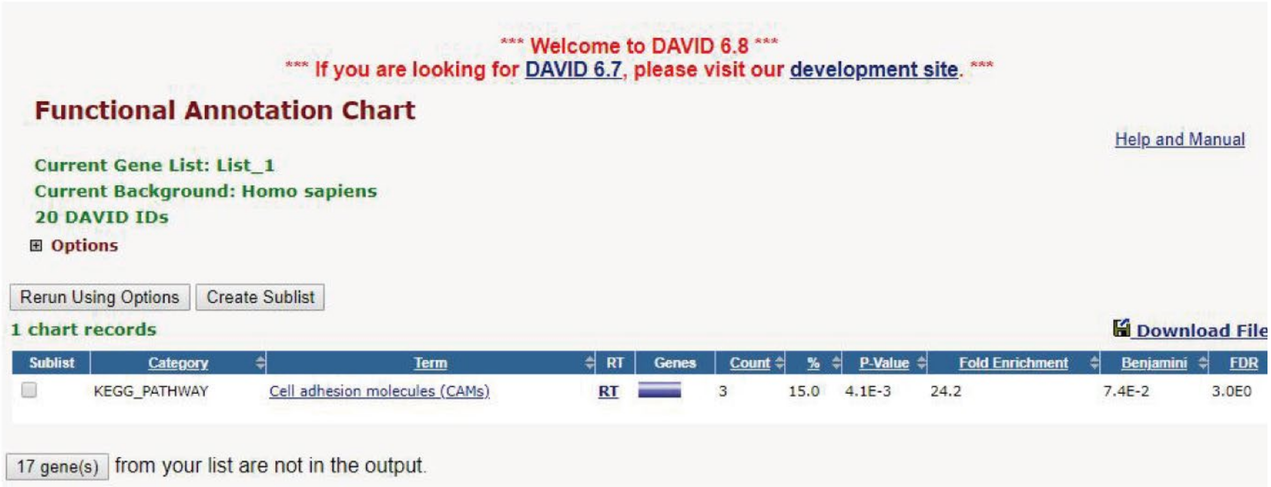

Figure S1. (**A**) Gene ontology (GO) analysis and significant enriched GO terms of 19 potential targeted mRNAs in HCC on their KEGG pathway using the online database DAVID 6.7.

Supplementary Table S1. The primer sequences used in this study were listed.

Name	Sequence 5'-3'
has-miR-3650-forward	GCAGAGGTGTGTCTGTAGAG
has-miR-3650-reverse	CAGTGCGTGTCGTGGAGT
U6-forward	CTCGCTTCGGCAGCACA
U6-reverse	AACGCTTCACGAATTTGCGT
NTNG1-forward	AAGTTTGGGATTACATGGCCTG
NTNG1-reverse	CGGAGGATCGAGTTTCACTTTC
NEGR1-forward	GGGAGGTGATAAGTGGTCAGT
NEGR1-reverse	CTGGGTGTATGTTGAGTCTGAAC
NAFSC-forward	CCAGACATCGCATGGTACAAG
NAFSC-reverse	TGATACGCAGGGCCTTATTAAAG
GAPDH-forward	CATCACCATCTTCCAGGAGCG
GAPDH-reverse	TGACCTTGCCCACAGCCTTG
